# Editorial: The newer paradigms in hypertension research and management

**DOI:** 10.3389/fmed.2026.1781466

**Published:** 2026-01-23

**Authors:** Komal Marwaha, Keith Norris, Freny Vagaiwalla Mody

**Affiliations:** 1 College of Medicine, University of Illinois, Peoria, IL, United States; 2 Department of Medicine, David Geffen School of Medicine, University of California, Los Angeles, Los Angeles, CA, United States; 3 Division of Cardiology, Veterans Affairs Greater Los Angeles Health Care System, Los Angeles, CA, United States

**Keywords:** age of hypertension onset shapes long-term renal risk, chronotherapy in hypertension treatment, recurrent hypoglycemia triggered secondary hypertension, nasal turbinate lymphatic obstruction in the pathogenesis of essential hypertension, impact of cuff placement on blood pressure readings, lay advisor-led interventions in hypertension management, elevated hemoglobin an independent predictor of hypertension, hypertension knowledge and adherence among primary care patients

Hypertension remains a major global health challenge, affecting more than 1.2 billion individuals worldwide and contributing to substantial cardiovascular morbidity and premature mortality (1). Despite decades of epidemiological insight and therapeutic innovation, population-level control remains inadequate: fewer than one in five individuals with hypertension achieve guideline-based targets (2). In addition to underdiagnosis and therapeutic inertia, poor adherence, especially among socioeconomically disadvantaged populations continues to limit effective management (3, 4). These persistent gaps underscore the need for fresh perspectives that extend beyond conventional risk stratification and pharmacologic control.

The articles included in this Research Topic collectively reflect the diverse and evolving paradigms shaping contemporary hypertension research. They examine the condition across multiple dimensions, temporal and mechanistic insights, circadian pharmacology, emerging neurovascular hypotheses, precision in measurement, population-specific determinants, and community- and system-level interventions. Together, these contributions offer a holistic view of hypertension as a complex interplay of biological, behavioral, and structural factors, while pointing toward novel strategies for prevention, diagnosis, and management.

At the temporal and mechanistic level, Qiu and Wu analyzed NHANES data to examine how the age of hypertension onset shapes long-term renal risk. Their cross-sectional study demonstrated that individuals diagnosed with hypertension before the age of 35 had more than double the risk of developing chronic kidney disease compared with normotensive peers. This finding underscores that hypertension is not simply a hemodynamic state but a reflection of progressive vascular burden that begins early, highlighting the need for screening for not only blood pressure, but end-organ changes in younger adults in the second decade with recommendations for lifestyle intervention and aggressive early therapeutic intervention to reduce vascular effects. Complementing this, Tsige and Ayele revisited malignant hypertension, one of the most severe clinical presentations. Their review emphasized current guideline-based treatment, endorsing intravenous labetalol and nicardipine as first-line agents, while stressing controlled reduction to avoid ischemic complications. Together, these studies span the disease's temporal extremes, from silent early onset to life-threatening crises and emphasize the necessity of timely detection and evidence-based management.

Building on these clinical insights, Nadeem et al. explored chronotherapy through a meta-analysis comparing morning vs. bedtime low-dose aspirin administration. Across more than 1,300 participants, bedtime dosing produced greater reductions in both systolic and diastolic blood pressure compared to morning dosing. These results highlight the interplay between circadian biology, vascular tone, and pharmacologic response, offering a low-cost, personalized strategy that leverages biological rhythms to optimize therapeutic outcomes.

Beyond established mechanisms, several contributions challenge conventional paradigms and open new investigative avenues. Mijalkovic and Sacic proposed that pancreatic insulinoma–induced recurrent hypoglycemia may trigger secondary hypertension through catecholamine surges and renal sodium retention. This opinion draws attention to neuroendocrine factors that may underlie secondary forms of hypertension. And additional novel pathobiological mechanism for secondary hypertension, with potential reversible management was proposed by Phillips and Schwartz, They advanced an innovative hypothesis implicating nasal turbinate lymphatic obstruction in the pathogenesis of essential hypertension. Drawing on nuclear imaging, they propose that nasal turbinate vasodilation impedes cerebrospinal fluid drainage, increases intracranial pressure, and triggers compensatory hypertension via the Cushing mechanism. This “selfish brain” model offers a novel neurovascular perspective and suggests unconventional therapeutic targets focused on autonomic modulation and nasal lymphatic pathways.

Another critical dimension explored in this Research Topic relates to measurement precision. Zhu et al. examined the impact of sphygmomanometer and cuff placement on blood pressure readings. In a large methodological study, measurements taken at the elbow fossa were consistently lower than those at the upper arm by 3–4 mmHg for both systolic and diastolic pressures. Given that treatment effect in large clinical trials is frequently in this range of change in BP in mmHg, this reinforces the importance of standardized technique and adherence to validated protocols, especially in large-scale screening or telemonitoring programs where even small deviations can shift diagnostic and treatment thresholds.

Expanding outward to system-level approaches, Patil et al. conducted a comprehensive systematic review and meta-analysis of lay advisor–led interventions, evaluating their effectiveness and implementation using the (Reach, Effectiveness, Adoption, Implementation, and Maintenance) RE-AIM framework. High-intensity lay advisor programs achieved greater reductions in both systolic and diastolic blood pressures compared with low-intensity or control groups. However, the authors noted limited reporting on adoption, implementation, and maintenance, underscoring the need to embed such interventions within sustainable health systems to achieve lasting impact.

Population-specific studies further enrich this Research Topic. Hongyu et al. examined high-altitude populations in China and identified elevated hemoglobin as an independent predictor of both carotid intima–media thickness and hypertension, with a threshold of 131 g/L. Their findings shed light on how chronic hypoxia and hematological adaptations shape vascular remodeling and may inform both local interventions and insights relevant to sea-level populations. Similarly, Al-Hazmi et al. conducted a cross-sectional study in eastern Saudi Arabia to assess hypertension knowledge and adherence among primary care patients. Only a small proportion of participants demonstrated high levels of knowledge, particularly regarding drug compliance, and knowledge was positively correlated with adherence. This emphasizes the critical role of culturally tailored patient education in improving long-term control. Finally, Hou et al. analyzed the cost drivers of hypertension hospitalizations in traditional Chinese medicine hospitals. They identified length of stay, age, and admission pathways as key factors and advocated integrating TCM's strengths in chronic disease management with standardized modern protocols to reduce economic burden and enhance efficiency.

Taken together, these contributions expand the landscape of hypertension research. From early onset risk and malignant hypertension to chronotherapy, neurovascular hypotheses, measurement precision, community engagement, and population-specific insights, they exemplify a shift from reductionist approaches toward integrated frameworks linking biology, behavior, and systems. Future progress will depend on bridging mechanistic discovery with clinical implementation, leveraging digital technologies, addressing sociocultural barriers, and personalizing care across diverse populations. The research presented in The Newer Paradigms in Hypertension Research and Management represents an important step toward redefining how hypertension is understood and managed in the coming decades.
